# Dynamic hematological changes in patients undergoing distal pancreatectomy with or without splenectomy: a population-based cohort study

**DOI:** 10.1186/s12893-020-00931-4

**Published:** 2020-10-31

**Authors:** Ming Cui, Jing-Kai Liu, Bang Zheng, Qiao-Fei Liu, Lu Zhang, Li Zhang, Jun-Chao Guo, Meng-Hua Dai, Tai-Ping Zhang, Quan Liao

**Affiliations:** 1 Department of General Surgery, Peking Union Medical College Hospital, Chinese Academy of Medical Sciences and Peking Union Medical College, Beijing, 100730 China; 2 School of Public Health, Faculty of Medicine, Imperial College London, London, W6 8RP UK; 3 Department of Hematology, Peking Union Medical College Hospital, Chinese Academy of Medical Sciences and Peking Union Medical College, Beijing, 100730 China; 4 Department of Clinical Laboratory, Peking Union Medical College Hospital, Chinese Academy of Medical Sciences and Peking Union Medical College, Beijing, 100730 China

**Keywords:** Distal pancreatectomy, Splenectomy, Spleen preservation, Leukocyte, Platelet

## Abstract

**Background:**

The clinical outcomes of patients who received distal pancreatectomy with splenectomy (DPS) and spleen-preserving distal pancreatectomy (SPDP) have been generally investigated. However, postoperative hematological changes after distal pancreatectomy with or without splenectomy are poorly understood.

**Methods:**

Information from patients undergoing distal pancreatectomy (DP) between January 2014 and June 2019 at a single institution was reviewed. A linear mixed-effects model was used to compare dynamic hematological changes between different groups.

**Results:**

A total of 302 patients who underwent DP were enrolled. In the long term, most postoperative hematological parameters remained significantly higher than preoperative levels in the DPS group, while postoperative lymphocyte, monocyte, basophil, and platelet levels returned to preoperative levels in the SPDP group. All postoperative hematological parameters except for red blood cell count and serum hemoglobulin level were significantly higher in the DPS group than in the SPDP group. There were no significant differences in hematological changes between the splenic vessel preservation (SVP) and Warshaw technique (WT) groups.

**Conclusions:**

Postoperative hematological changes were significantly different between the DPS and SPDP groups. Compared to DPS, SPDP reduced abnormal hematological changes caused by splenectomy. SVP and WT were comparable in terms of postoperative hematological changes.

## Background

Distal pancreatectomy (DP) is the standard operation for lesions located at the body or tail (left side) of the pancreas. Splenectomy is often involved in DP due to anatomical proximity and the shared principal vessels between the spleen and left pancreas. To reduce the risks associated with removing the spleen, which functions as a hematologic and immunological organ, spleen-preserving distal pancreatectomy (SPDP) has been established, including two major methods: the conventional splenic vessel preservation (SVP) technique and the short gastric vessel-preserving technique, or the Warshaw technique (WT) [1–3]. Recently, SPDP has been increasingly applied among patients with benign or low-grade pancreatic lesions [4, 5].

Various studies have explored the effects of splenectomy. Previous studies indicate that splenectomy is associated with several complications during follow-up, such as infections, thromboembolism, and increased risk of developing cancer [6–8]. Many pancreatic surgeons further compared the clinical outcomes between patients who received distal pancreatectomy with splenectomy (DPS) and SPDP, and the results showed that the SPDP group may present fewer infection complications, less operative blood loss, and a lower overall morbidity rate [9, 10]. To understand the mechanisms of the above aberrations, several studies have examined hematologic and immunological changes after splenectomy, presenting thrombocytosis, leukocytosis, decreased immunoglobulin M production, and depressed phagocytic activity [11–14]. The majority of these studies, however, were based on patients with existing hematological diseases or severe trauma, probably with selection bias as a result of the influences of hematological disorders, massive blood loss on peripheral hematological system, or surgery itself. In addition, data on dynamic changes in different peripheral blood cell populations after splenectomy are still lacking in previous studies. Exploring the above dynamic changes might be helpful to further understand the pathophysiological processes after DP with or without splenectomy.

The aim of this study was to identify dynamic hematological changes in patients receiving DPS or SPDP, as well as differences between two spleen-preserving techniques (SVP or WT), to explore hematological changes after DP in a population-based cohort study.

## Methods

### Patients

Between January 2014 and June 2019, consecutive patients who underwent DPS or SPDP and were recorded in the retrospective database at Peking Union Medical College Hospital were considered suitable for the study. Considering that SPDP is not indicated for malignant lesions of the pancreas, patients with a postoperative diagnosis of benign or low-grade malignant lesions were extracted. To avoid interference caused by perioperative hematological abnormalities, patients who presented with the following conditions were excluded: age less than 18 years, abnormal preoperative peripheral blood cell counts, intraoperative blood loss more than 400 ml, intraoperative or postoperative blood transfusion, and severe postoperative complications, including infections, bleeding, or perioperative death. No patient was vaccinated perioperatively. This study was approved by the Institutional Review Board of Peking Union Medical College Hospital (S-K832). The need for informed consent was waived because this was a retrospective analysis of data from the hospital database.

### Surgical techniques

Surgical procedures applied for the enrolled patients included DPS, SPDP with SVP, and SPDP with WT. The surgical procedures were standardized, and all surgeons had received similar training. The protocols for all of the surgical procedures have been previously described [1, 15, 16]. Briefly, the pancreas was transected using an endoscopic linear stapler or energy-based devices. For DPS, the spleen was mobilized to be removed en-block with the pancreas. For SPDP with SVP, the splenic vessels were dissected and preserved from the pancreatic body to the tail. For SPDP with WT, the short gastric vessels and left gastroepiploic vessels were carefully protected before dividing the splenic vessels and the pancreas.

### Laboratory tests

The values of leukocytes (including white blood cells (WBCs), neutrophils, lymphocytes, monocytes, eosinophils, and basophils), platelets, red blood cells (RBCs), and serum hemoglobin were determined before surgery and on postoperative days (PODs) 1, 3, 5, and 7; postoperative week (POW) 2 (ranges from 8 to 27 days, median 13 days); and postoperative months (POMs) 1 (ranges from 1 to 3 months, median 1.3 months) and 3 (ranges from 3 to 44 months, median 8.1 months). All these parameters were evaluated routinely in the clinical laboratories of the Peking Union Medical College Hospital.

### Statistical analysis

The clinical characteristics of patients were compared between the DPS and SPDP groups, with t tests for continuous variables and chi-square tests for categorical variables. Dynamic changes in the nine peripheral blood cell populations were examined using paired-samples t tests that compared the measurements at each postoperative time point with the preoperative values separately for patients in the DPS group and the SPDP group. Differences in dynamic changes between these two groups were then tested using a linear mixed-effects model, with levels of peripheral blood cell populations as dependent variables. A random effect for subject-level intercepts was used to account for person-specific differences in blood measurements. Fixed effects included a time variable (eight timepoints) and surgical group (DPS or SPDP), with further adjustment for age, sex, pathological diagnosis and surgical techniques (open or laparoscopic). Interactions between time and surgical group were tested to determine the between-group differences in hematological dynamic changes. We also compared hematological dynamic changes between patients receiving different spleen-preserving techniques (SVP versus WT) with similar modeling procedures.

In addition, we further explored the influences of age, sex, and surgical techniques (open or laparoscopic) on hematological dynamic changes in patients receiving DPS. Interactions between time and the potential moderator were tested in linear mixed-effects models within the DPS group. Sensitivity analyses were conducted by excluding potential outliers of blood measurements [beyond the mean ± 3 standard deviations (SDs)] at each timepoint and then repeating the main analyses, and additionally adjusting for intraoperative blood loss to account for possible residual confounding bias in between-group comparisons.

Statistical analysis was performed using SPSS version 24 (SPSS Inc., Chicago, IL, USA) and STATA 14 (StataCorp, College Station, TX, USA). Where applicable, a *P* value of less than 0.05 was considered statistically significant.

## Results

### Patient characteristics

During the study period, a total of 815 patients who underwent DPS or SPDP were identified retrospectively, of whom 513 patients were excluded according to the prespecified criteria. Of the 302 enrolled patients, 169 patients underwent DPS, and 133 patients underwent SPDP (see Additional file 1, Fig. S1). Among the 302 patients, there were 87 (28.8%), 70 (23.2%), 65 (21.5%), 59 (19.5%), 13 (4.3%), and 8 (2.6%) cases of solid pseudopapillary tumor (SPT), pancreatic neuro-endocrine tumor (PNET), mucinous cystic neoplasm (MCN), serous cystic neoplasm (SCN), intraductal papillary mucinous neoplasm (IPMN), and other tumors, respectively. Table 1 describes the characteristics of the DPS group versus those of the SPDP group. Compared to the DPS group, the SPDP group was slightly younger (41.8 versus 47.3 years; *P* = 0.004). The pathological diagnoses were significantly different between the two groups (*P* = 0.012). To avoid the potential confounding bias caused by different patient characteristics between these two groups, linear mixed-effects models were used with adjustment for these covariates in the following analyses.

**Table 1:** Patient characteristics

Characteristics	DPS	SPDP	*P* value
Sample size, n	169	133	
Sex (Female:Male)	123:46	109:24	0.061
Age, mean (SD), years	47.3 (15.8)	41.8 (13.1)	0.004
Pathological diagnosis, n (%)			0.011
SPT	38 (22.5)	49 (36.8)	
PNET	41 (24.3)	29 (21.8)	
MCN	42 (24.9)	23 (17.3)	
SCN	31 (18.3)	28 (21.1)	
IPMN	12 (7.1%)	1 (0.8)	
Other tumors	5 (3.0)	3 (2.3)	
Surgical techniques, n (%)			< 0.001
Open	44 (26.0)	9 (6.8)	
Laparoscopic	125 (74.0)	124 (93.2)	
Intraoperative blood loss, mean (SD), mL	166.8 ± 126.9	111.2 ± 105.1	< 0.001

*DPS* distal pancreatectomy with splenectomy, *SPDP* spleen-preserving distal pancreatectomy, *SD* standard deviation, *SPT* solid pseudopapillary neoplasm, *PNET* pancreatic neuroendocrine tumor, *MCN* mucinous cystic neoplasm, *SCN* serous cystic neoplasm, *IPMN* intraductal papillary mucinous neoplasm

### Effect of DP on dynamic changes in peripheral blood cell populations

**Fig. 1:**
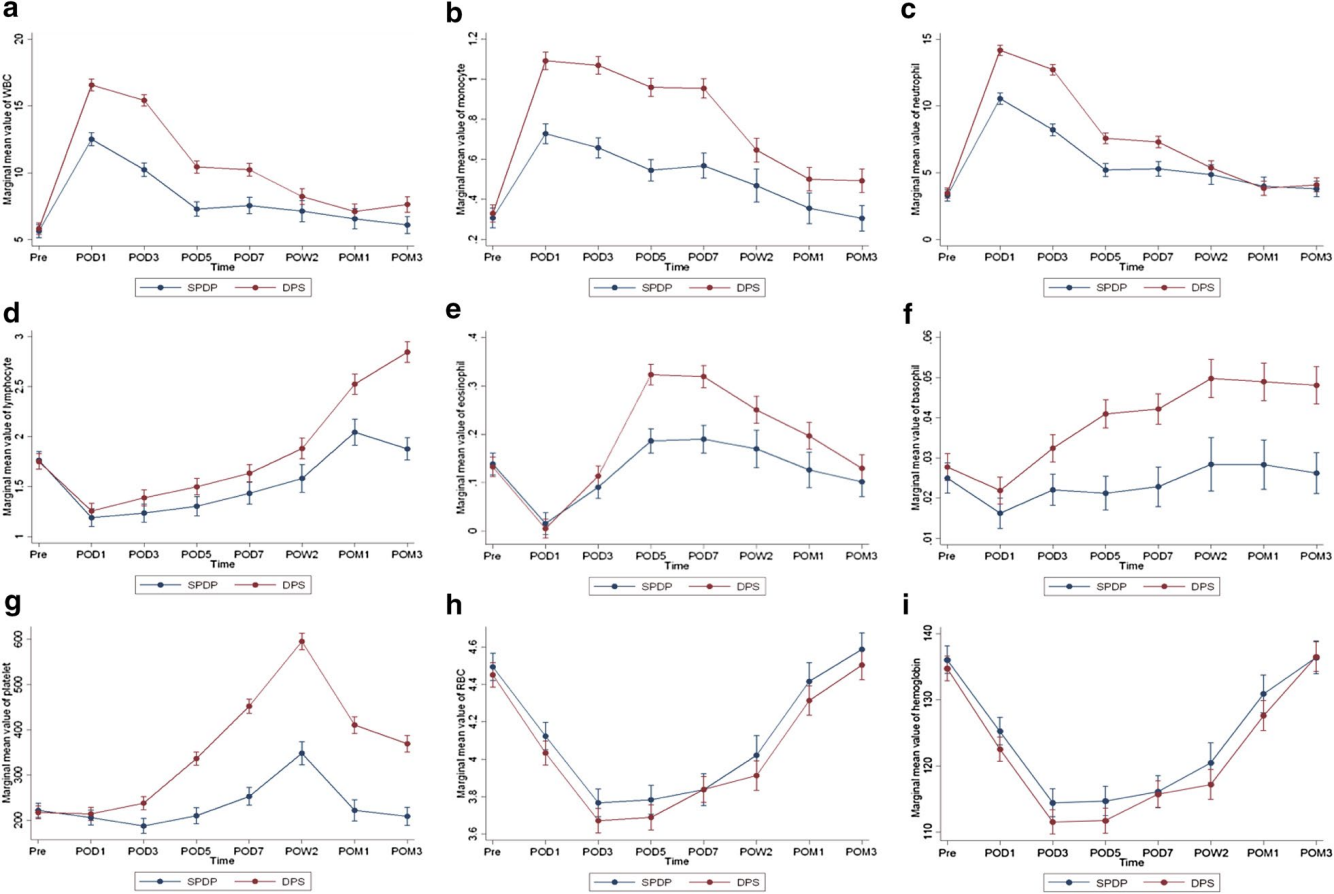
Longitudinal changes in peripheral blood cell populations in the DPS group vs. SPDP group. **a** WBC, **b** neutrophil, **c** monocyte, **d** lymphocyte, **e** eosinophil, **f** basophil, **g** platelet, **h** RBC, **i** hemoglobin

The DPS and SPDP groups generally presented similar patterns of hematological changes after surgery (Table 2). Peripheral WBC counts, neutrophil counts, and monocyte counts were significantly elevated on POD1 and gradually returned to their original values since POD3 (Fig. 1a–c). Lymphocyte counts, eosinophil counts, and basophil counts were significantly reduced on POD1 and gradually recovered since POD3 (Fig. 1d–f). Platelet counts of both the DPS group and the SPDP group peaked at POW2 and began returning to their normal values after that point (Fig. 1g). RBC counts and serum hemoglobin levels continued to decrease through POD5 and began to recover after POD7 (Fig. 1h, i). From a long-term perspective, the levels of most of the parameters, including WBC counts, neutrophil counts, lymphocyte counts, monocyte counts, basophil counts, and platelet counts, were still significantly higher than the preoperative levels in the DPS group at POM3 (*P* < 0.05). However, in the SPDP group, lymphocyte counts, monocyte counts, basophil counts, and platelet counts returned to preoperative levels at POM3 (*P* > 0.05). RBC counts and serum hemoglobin levels returned to preoperative levels at POM3 in both the DPS and SPDP groups (*P* > 0.05) (Table 2).

**Table 2:** Mean levels of blood cell populations of patients in the DPS and SPDP groups

Blood cell populations	Time points							
	Preoperative (n = 302)	POD1 (n = 301)	POD3 (n = 284)	POD5 (n = 247)	POD7 (n = 193)	POW2 (n = 111)	POM1 (n = 120)	POM3 (n = 143)
WBCs (10^9^/L)								
DPS	5.82	16.58**	15.44**	10.41**	10.23**	8.23**	7.23**	7.51**
SPDP	5.64	12.51**	10.23**	7.35**	7.59**	7.31**	6.62**	6.26**
Neutrophils (10^9^/L)								
DPS	3.50	14.19**	12.74**	7.58**	7.32**	5.39**	3.96*	3.99**
SPDP	3.30	10.54**	8.21**	5.27*	5.31*	5.01	4.00**	3.92**
Lymphocytes (10^9^/L)								
DPS	1.75	1.26**	1.39**	1.49**	1.62**	1.84	2.50**	2.85**
SPDP	1.77	1.20**	1.25**	1.30**	1.46**	1.59	2.09*	1.89
Monocytes (10^9^/L)								
DPS	0.32	1.09**	1.07**	0.95**	0.95**	0.65**	0.50**	0.47**
SPDP	0.32	0.74**	0.67**	0.55**	0.59**	0.50**	0.37*	0.33
Eosinophils (10^9^/L)								
DPS	0.14	0.01**	0.12*	0.32**	0.32**	0.25**	0.21**	0.14
SPDP	0.13	0.01**	0.08**	0.18**	0.18**	0.16**	0.13	0.10**
Basophils (10^9^/L)								
DPS	0.028	0.022**	0.032**	0.040**	0.042**	0.049**	0.050**	0.048**
SPDP	0.025	0.016**	0.022	0.021*	0.022	0.030	0.030	0.027
Platelets (10^9^/L)								
DPS	213	210	233**	328**	442**	581**	410**	356**
SPDP	228	212**	194**	219*	261**	355**	238	217
RBC (10^12^/L)								
DPS	4.46	4.04**	3.68**	3.68**	3.84**	3.91**	4.29**	4.53
SPDP	4.48	4.11**	3.75**	3.74**	3.77**	4.05**	4.35	4.56
Serum hemoglobin (g/L)								
DPS	135	123**	112**	112**	116**	117**	127**	138
SPDP	135	125**	114**	113**	113**	121**	129*	136

*DPS* distal pancreatectomy with splenectomy, *SPDP* spleen-preserving distal pancreatectomy, *POD* postoperative day, *POW* postoperative week, *POM* postoperative month, *WBC* white blood cell, *RBC* red blood cell

*P < 0.05, **P < 0.01, using paired t test comparing postoperative time points with the preoperative level within the DPS group and the SPDP group separately

### Comparison of dynamic changes in peripheral blood cell populations between the DPS group and SPDP group

No differences in preoperative levels of the nine peripheral blood cell populations were identified between the DPS and SPDP groups after adjusting for baseline characteristics (*P* > 0.05), indicating that the two groups were comparable before surgery. Although the overall patterns of hematological changes after surgery were similar in the two groups, differences in the magnitude of the dynamic changes between groups were observed. Generally, the postoperative levels of almost all parameters of peripheral blood cell populations, except for RBC counts and serum hemoglobin levels, were significantly higher in the DPS group than in the SPDP group (*P* < 0.05) (Fig. 1). The differences in the WBC counts, neutrophil counts, and monocyte counts between the DPS and SPDP groups peaked between POD1 and POD3 and gradually decreased over time (Fig. 1a–c). The differences in lymphocyte counts, basophil counts, and platelet counts between the DPS and SPDP groups continued to increase during the whole follow-up period (Fig. 1d, f, g). Regarding eosinophil counts, the differences in the values between the two groups increased until POD7 and then decreased at the later follow-ups (Fig. 1e). The RBC counts and serum hemoglobin levels were not significantly different during the whole follow-up period between these two groups (*P* > 0.05) (Fig. 1h, i). From a long-term perspective, the WBC counts, monocyte counts, lymphocyte counts, basophil counts, and platelet counts were significantly higher in the DPS group than in the SPDP group at POM3 (*P* < 0.05), while the levels of neutrophil counts, eosinophil counts, RBC counts, and serum hemoglobin levels at POM3 were not significantly different between the DPS and SPDP groups (*P* > 0.05) (Fig. 1).

### Comparison of dynamic changes in peripheral blood cell populations between the SVP and WT groups

In the SPDP group, 71 patients received SPDP with SVP, and 62 patients received SPDP with WT. We further compared dynamic changes in peripheral blood cell populations between the SVP and WT groups to study the effects of different spleen-preserving techniques. The results showed that postoperative hematological changes in all nine parameters of peripheral blood populations had no significant differences between the SVP group and the WT group from both short-term and long-term perspectives after surgery (*P* > 0.05, see Additional file 2, Fig. S2). The results remained similar in sensitivity analyses after excluding potential hematological parameter outliers and additionally adjusting for intraoperative blood loss.

### Moderating effects of patient characteristics on dynamic changes in peripheral blood cell populations in the DPS group

**Fig. 2:**
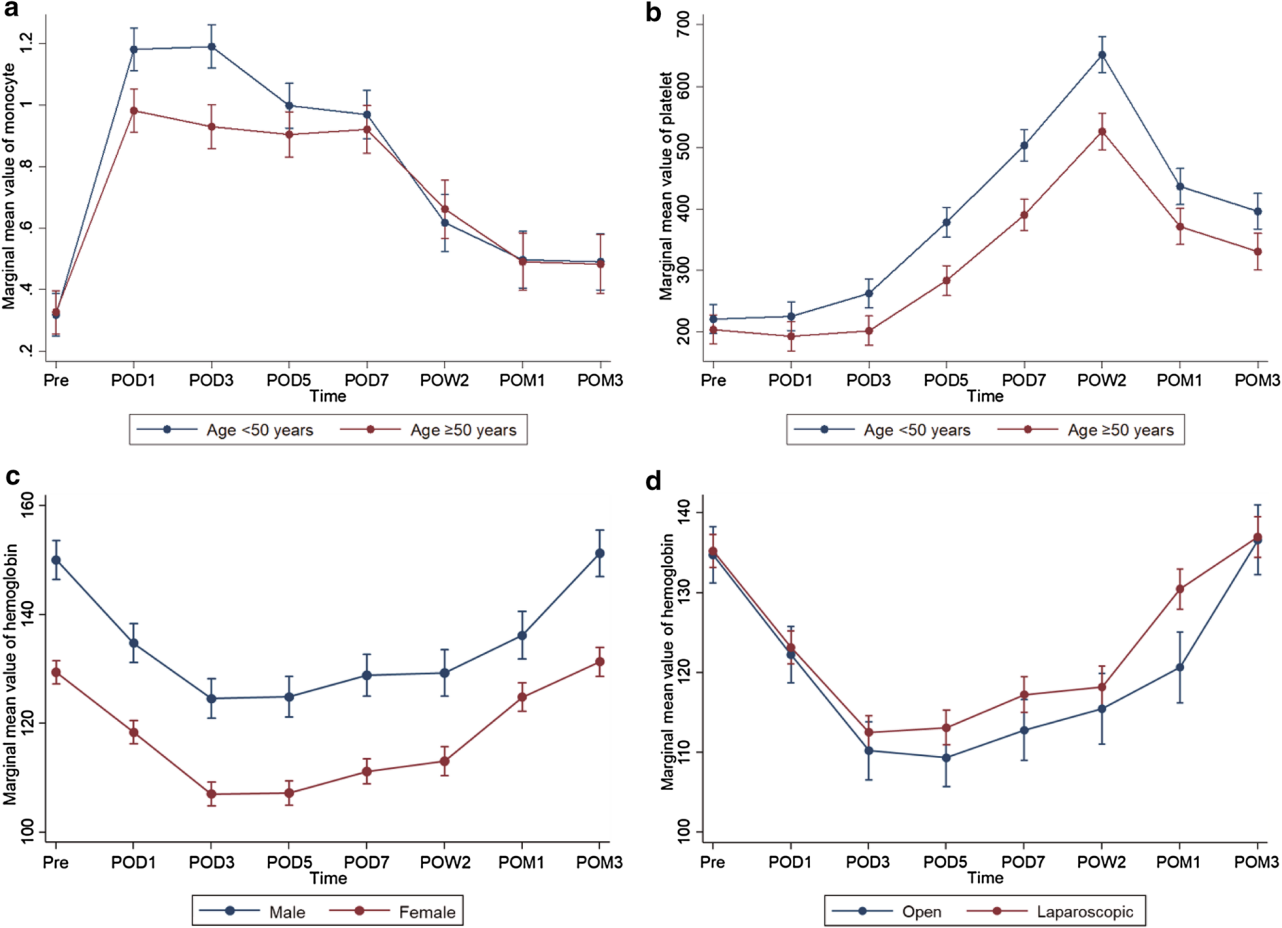
Moderating effects of patient characteristics on longitudinal changes in peripheral blood cell populations

We further analyzed the moderating effects of demographic and clinical characteristics on dynamic changes in peripheral blood cell populations in the DPS group. Monocyte counts and platelet counts were elevated to significantly higher levels in patients younger than 50 years than in patients older than 50 years at POD1 and POD3 (*P* < 0.05) (Fig. 2a, b). Serum hemoglobin levels decreased faster and recovered slower in male patients than in female patients (*P* < 0.05) (Fig. 2c). In addition, serum hemoglobin levels recovered faster in the patients undergoing laparoscopic surgery than in the patients undergoing open surgery (*P* < 0.05) (Fig. 2d). Other unmentioned parameters of peripheral blood cell populations were not significantly affected by age, sex, or surgical techniques.

## Discussion

The present study precisely described the whole process of dynamic hematological changes after DPS or SPDP. Compared to previous studies of splenectomy, the present study has two main advantages. First, the enrolled patients did not present any evident preoperative aberrations of the peripheral hematological system. For these patients, splenectomy was a forced intraoperative decision to ensure the safety of the operations rather than a therapeutic method. Second, the SPDP group is an ideal control for the DPS group to specifically explore the effects of splenectomy with a relatively large number of patients enrolled. As such, this study could largely avoid the interference of underlying hematological diseases and the influence of the effect of surgery per se on perioperative peripheral blood cell populations.

Based on our results, the DPS and SPDP groups presented similar patterns of hematological changes after DP. Platelet counts of both the DPS and SPDP groups were elevated and peaked at POW2, which is consistent with previous studies [17, 18]. Almost all peripheral blood cell populations were significantly higher in the DPS group than in the SPDP group. From a short-term perspective, the differences in WBC counts, neutrophil counts, and monocyte counts peaked at POD1 or POD3 and then decreased with time, while the differences in lymphocyte counts, basophil counts, and platelet counts continued to increase during follow-up. From a long-term perspective, peripheral blood populations in the SPDP group were closer to preoperative levels than those in the DPS group, indicating that SPDP was helpful to avoid pathophysiological responses caused by splenectomy. In addition, lymphocyte counts, monocyte counts, basophil counts, and platelet counts remained significantly higher in the DPS group than in the SPDP group, indicating the continuous long-term effect of splenectomy on the abovementioned blood cell populations. The mechanism for this is still unclear. Furthermore, we compared dynamic changes in peripheral blood cell populations between the SVP and WT groups for patients receiving SPDP, and the results did not show significant differences according to short-term and long-term perspectives postoperatively, indicating that different techniques of SPDP may not differ from each other in terms of hematological changes. This may partially explain the comparable clinical outcomes between patients undergoing SVP and those undergoing WT [19].

Distal pancreatectomy, including DPS and SPDP, is a high-risk abdominal surgery that is often associated with several postoperative complications, such as pancreatic fistula, infections, or bleeding. Peripheral blood examination is routinely performed after surgery and is one of the most convenient tests to monitor the recovery of patients and identify postoperative complications early. A better understanding of dynamic hematological changes after DPS and SPDP is important to differentiate normal postoperative responses from postoperative complications among these patients. An elevated WBC count is often considered an indicator of infection, while leukocytosis is also a physiologic process that occurs after splenectomy. Several studies have reported that postoperative WBC counts should be adjusted to evaluate the presence of infections among patients undergoing splenectomy [20, 21]. Maatman et al. [22] who retrospectively reviewed 158 patients undergoing DPS reported that POD3 WBC counts above 16 × 10^9^/L or an increase in WBC counts greater than 9 × 10^9^/L from preoperative baseline was associated with major morbidity. In our study, the results were similar: the average POD3 WBC count was 15.4 × 10^9^/L, and the increase from baseline was 9.6 × 10^9^/L in the DPS group. In addition, the average POD3 WBC count was 10.2 × 10^9^/L, and the increase from baseline was 4.6 × 10^9/^L in the SPDP group. Furthermore, monitoring postoperative changes in serum hemoglobin levels and RBC counts is a direct way to evaluate the occurrence of postoperative bleeding. From our results, both serum hemoglobin levels and RBC counts decreased and reached a minimum from POD3 to POD5. The average minimum values of serum hemoglobin levels and RBC counts were not significantly different between the DPS (112 g/L, 3.68 × 10^12^/L, respectively) and SPDP groups (113 g/L, 3.74 × 10^12^/L, respectively), which indicated that splenectomy may not influence the clinical evaluation of postoperative bleeding or hematopoietic functions among patients undergoing DP.

Exploring the effect of the spleen, particularly the effect of splenectomy, on tumorigenesis is a hot topic, but no firm conclusion has been drawn yet. Of note, the spleen plays vital roles in host innate and adaptive immunity, which could be potential mechanisms influencing tumor growth. Previous epidemiological studies have indicated that preoperative cancer-free patients would suffer an increased cancer risk after splenectomy during long-term follow-up [23]. However, the effect of splenectomy on tumor progression in cancer patients might be another issue. Several clinical studies have investigated the impact of splenectomy on long-term survival among patients with different types of cancers, but the results are paradoxical [24–26]. The mechanisms of the effect of the spleen on tumorigenesis are inconclusive. Prehn [27] proposed that whether splenectomy enhanced or inhibited tumor progression might depend on the spleen to tumor ratio. Stöth et al. [28] revealed that splenectomy limited the infiltration of immune-suppressing cells and suppressed the metastasis of breast cancer. In contrast, pancreatic cancer exhibited more aggressive growth and excessive peritoneal seeding as well as a decreased ratio of cytotoxic T cells to FoxP3 + Treg cells after splenectomy [29]. In our study, splenectomy significantly affected peripheral blood cell populations in patients undergoing DP. Several studies have reported that peripheral blood cell populations, including WBC counts, granulocyte counts, and monocyte counts, were associated with the prognosis of patients with pancreatic cancer [30, 31]. As such, the dynamic changes in peripheral blood cell populations after splenectomy might be part of the potential mechanisms to explain altered host immune functions and clinical prognosis. Conventionally, DPS is considered the reference operation for left-sided pancreatic cancer to obtain a negative surgical margin. Recently, Navez et al. [32] and Collard et al. [33] reported that direct splenic involvement was not common in left-sided pancreatic cancers, especially pancreatic body cancers. Perhaps we should consider the decision of splenectomy much more from a biological perspective rather than a surgical perspective. More clinical and basic studies focusing on the oncologic effects of splenectomy on pancreatic cancer will help address whether splenectomy should be routinely performed for patients with left-sided pancreatic cancer [34].

There are several limitations to the present study. First, the data that we analyzed were limited to parameters included in routine clinical laboratory tests. With novel techniques such as flow cytometry and single-cell sequencing, we could prospectively obtain more information on dynamic changes in immune cell subsets. In addition, there were some missing blood measurement values at postoperative time points; thus, a linear mixed-effects model was adopted in the repeated-measures analysis, which could make use of the available information at all time points to conserve statistical power.

## Conclusions

In conclusion, our study described the dynamic hematological changes in patients undergoing DP with or without splenectomy. Compared to DPS, SPDP was helpful to avoid abnormal hematological changes caused by splenectomy. The two spleen-preserving techniques (SVP and WT) were comparable in terms of postoperative hematological changes. These findings might be helpful for the clinical management of patients who undergo DP and may pave the way for a deeper understanding of the pathophysiological responses after splenectomy.

## Supplementary information

**Additional file 1: Fig. S1.** Flowchart showing patient enrolment in the present study. *DP* distal pancreatectomy, *DPS* distal pancreatectomy with splenectomy, *SPDP* spleen-preserving distal pancreatectomy.

**Additional file 2: Fig. S2.** Longitudinal changes in peripheral blood cell populations in the WT group vs. SVP group. **a** WBC, **b** neutrophil, **c** monocyte, **d** lymphocyte, **e** eosinophil, **f** basophil, **g** platelet, **h** RBC, **i** hemoglobin.

## Data Availability

The original data and materials are available from the corresponding author on reasonable request.

## Abbreviations

DP: Distal pancreatectomy
DPS: Distal pancreatectomy with splenectomy
SPDP: Spleen-preserving distal pancreatectomy
SVP: Splenic vessel preservation
WT: Warshaw technique
WBC: White blood cell
RBC: Red blood cell
POD: Postoperative day
POW: Postoperative week
POM: Postoperative month
SD: Standard deviation
SPT: Solid pseudopapillary tumor
PNET: Pancreatic neuro-endocrine tumor
MCN: Mucinous cystic neoplasm
SCN: Serous cystic neoplasm
IPMN: Intraductal papillary mucinous neoplasm
