# Supplementary figures and images for: Dynamic hematological changes in patients undergoing distal pancreatectomy with or without splenectomy: a population-based cohort study
Source: BMC Surg. 2020 Oct 31;20:265. doi: 10.1186/s12893-020-00931-4 (PMC7603722; doi:10.1186/s12893-020-00931-4)

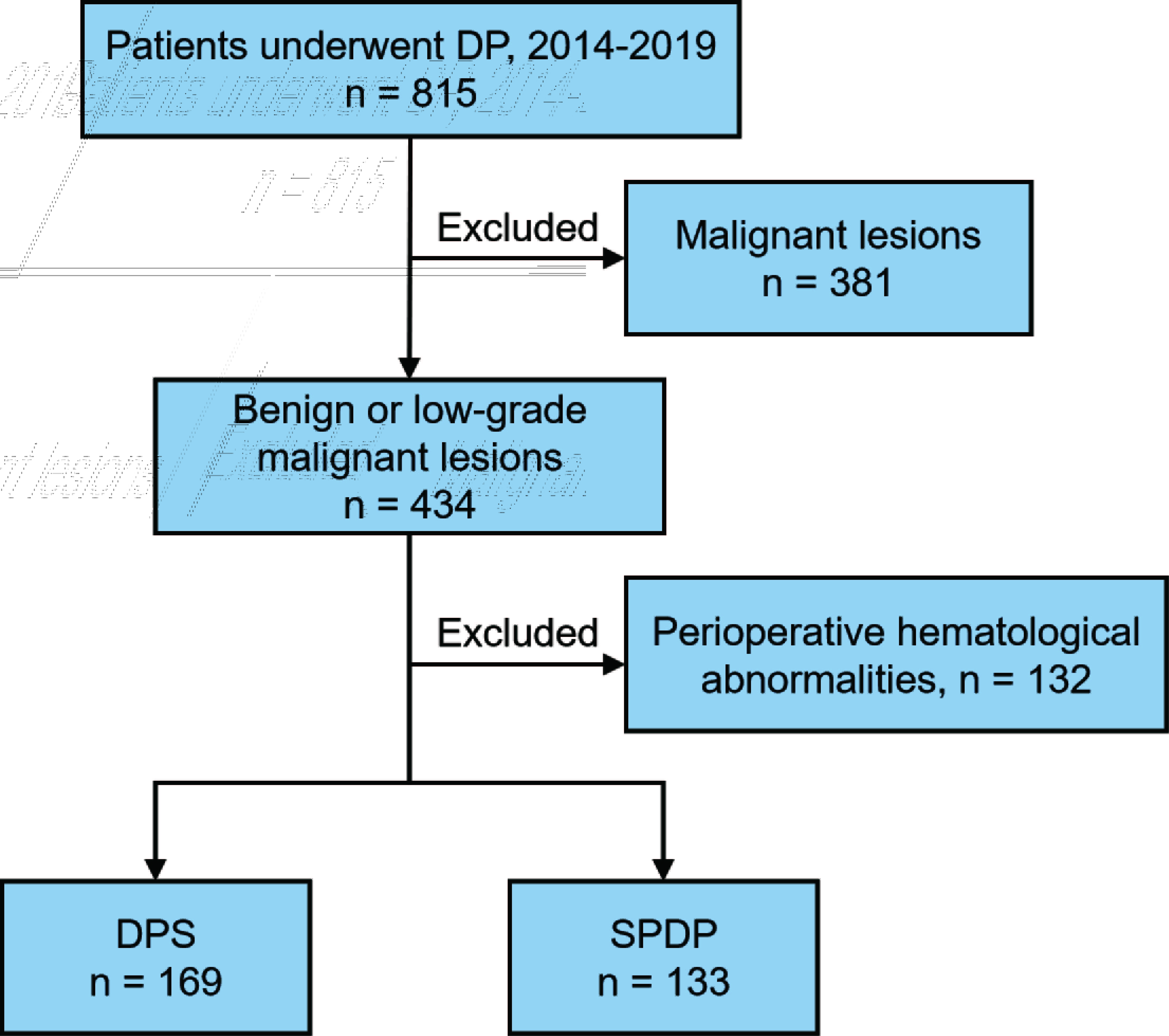

Supplement: Supplementary file 1 — Additional file 1: Fig. S1. Flowchart showing patient enrolment in the present study. DP distal pancreatectomy, DPS distal pancreatectomy with splenectomy, SPDP spleen-preserving distal pancreatectomy. [file 12893_2020_931_MOESM1_ESM.jpg]

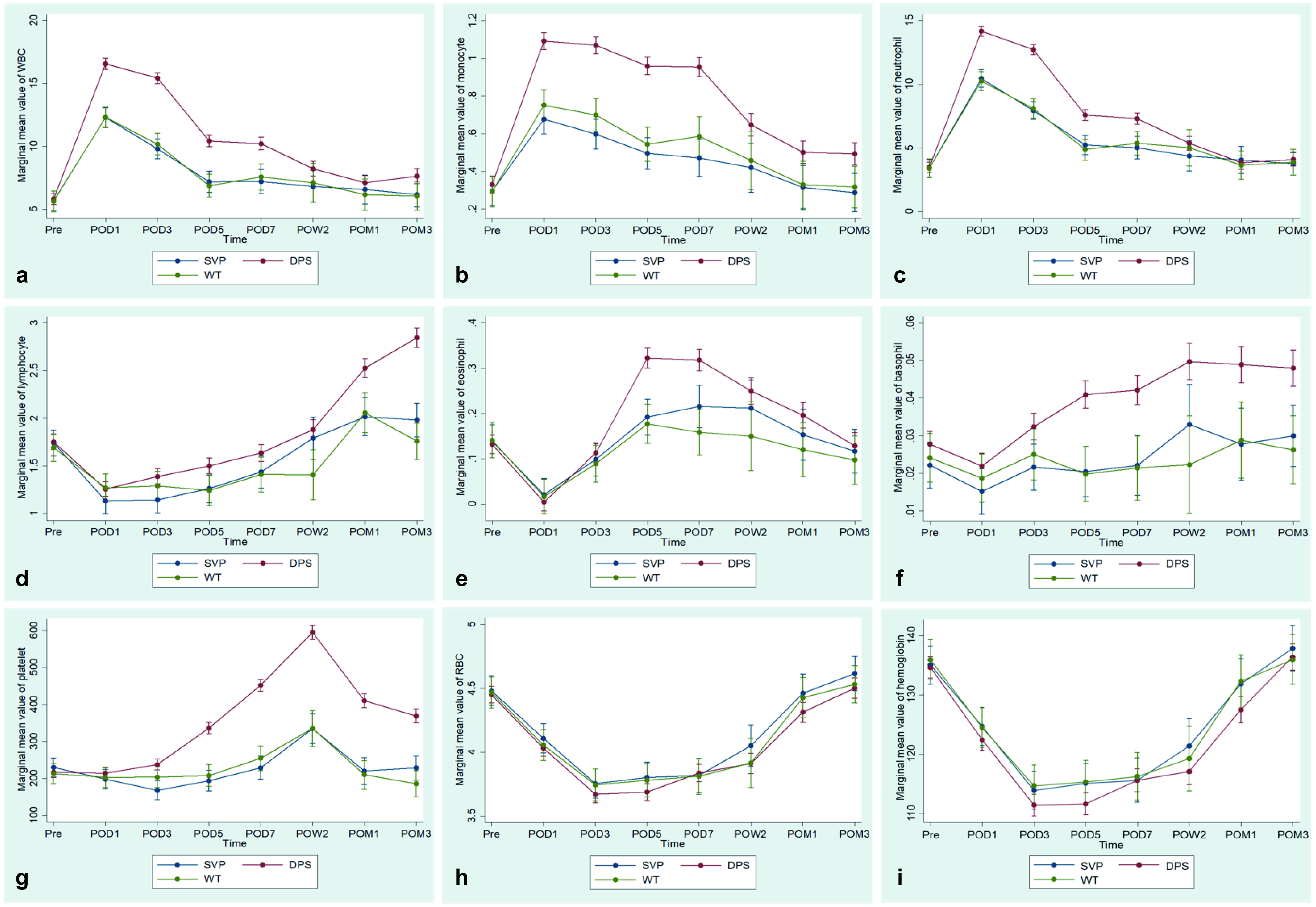

Supplement: Supplementary file 2 — Additional file 2: Fig. S2. Longitudinal changes in peripheral blood cell populations in the WT group vs. SVP group. a WBC, b neutrophil, c monocyte, d lymphocyte, e eosinophil, f basophil, g platelet, h RBC, i hemoglobin. [file 12893_2020_931_MOESM2_ESM.tif]
